# Exploring the causal and effect nature of EQ-5D dimensions: an application of confirmatory tetrad analysis and confirmatory factor analysis

**DOI:** 10.1186/s12955-018-0975-y

**Published:** 2018-07-31

**Authors:** Thor Gamst-Klaussen, Claire Gudex, Jan Abel Olsen

**Affiliations:** 1 Department of Community Medicine, UIT the Arctic University of Norway, 9016 Tromsø, Norway; 2 Department of Clinical Research, University of Southern Denmark, Odense, Denmark; 3 Division of Health Services, Norwegian Institute of Public Health, 0213 Oslo, Norway

**Keywords:** EQ-5D-5 L, Health outcomes, Confirmatory tetrad analysis, Preference weights, Causal indicators, Effect indicators

## Abstract

**Background:**

The relationship between the various items in an HRQoL instrument is a key aspect of interpreting and understanding preference weights. The aims of this paper were i) to use theoretical models of HRQoL to develop a conceptual framework for causal and effect relationships among the five dimensions of the EQ-5D instrument, and ii) to empirically test this framework.

**Methods:**

A conceptual framework depicts the symptom dimensions [Pain/discomfort (PD) and Anxiety/depression (AD)] as causal indicators that drive a change in the effect indicators of activity/participation [Mobility (MO), Self-care (SC) and Usual activities (UA)], where MO has an intermediate position between PD and the other two effect dimensions (SC and UA). Confirmatory tetrad analysis (CTA) and confirmatory factor analysis (CFA) were used to test this framework using EQ-5D-5L data from 7933 respondents in six countries, classified as healthy (*n* = 1760) or in one of seven disease groups (*n* = 6173).

**Results:**

CTA revealed the best fit for a model specifying SC and UA as effect indicators and PD, AD and MO as causal indicators. This was supported by CFA, revealing a satisfactory fit to the data: CFI = 0.992, TLI = 0.972, RMSEA = 0.075 (90% CI 0.062–0.088), and SRMR = 0.012.

**Conclusions:**

The EQ-5D appears to include both causal indicators (PD and AD) and effect indicators (SC and UA). Mobility played an intermediate role in our conceptual framework, being a cause of problems with Self-care and Usual activities, but also an effect of Pain/discomfort. However, the empirical analyses of our data suggest that Mobility is mostly a causal indicator.

## Background

Health-related quality of life (HRQoL) instruments comprise items that relate to various aspects of health and functioning. Previous research has attempted to classify the items included in these instruments as being causal or effect indicators of HRQoL [1]. Effect indicators (also called reflective indicators) can be seen as manifestations of an underlying construct. Thus, a change in the construct will lead to, or drive, a change in the effect indicators. In contrast, causal indicators (also called formative indicators) drive a change in the construct. There is evidence to suggest that symptoms have a strong causal component that drives a change in other items [2, 3]. The research into the causal nature of various HRQoL items has been limited to *disease-specific* instruments. No studies have investigated causal relationships in *generic* preference-based measures of HRQoL, commonly referred to as health state utility (HSU) instruments [4], which have an important role in cost-effectiveness analyses that are increasingly being used to aid policy decisions. Based on theoretical models, and methodological lessons from previous research, this paper seeks to fill a knowledge gap by identifying a causal pattern in the most widely applied HSU instrument, the EQ-5D [5–7]. The causal pattern of items in the cancer-specific EORTC QLQ-C30 instrument has been investigated in three studies. Using applied graphical methods and cross-tabulation of response frequencies, Fayers et al. found strong evidence that physiological symptom items (e.g. nausea, memory problems, shortness of breath) were causal, while items such as poor concentration, irritability, and feeling tense were likely to be effect indicators [2]. Boehmer and Luszczynska applied confirmatory factor analysis and found satisfactory fit for a model with both causal indicators (symptoms e.g. fatigue, pain) and effect indicators (e.g. physical, role, cognitive, social, and emotional functioning) [3]. It was noted that physical functioning and pain might be intermediate types of indicators. Using eight EORTC QLQ-C30 items, Bollen et al. provided an example of confirmatory tetrad analysis (CTA) and concluded that symptom items (e.g. shortness of breath, problems sleeping, lack of appetite) should be treated as causal indicators, while global health status and quality of life should be treated as effect indicators [8].

Factor analysis is a common psychometric approach to investigate the relationship between items and unobserved constructs, which is one technique in structural equation modelling (SEM) used for scale design and validation. However, factor analysis usually depends on a set of homogenous items and is often not appropriate if both causal and effect items are present [2]. However, other SEM techniques incorporate causal paths to model the relationship among different types of items [9, 10]. Confirmatory tetrad analysis may be the best empirical approach for determining if items should be treated as causal or effect indicators [8]. This paper is the first to apply CTA in HSU instruments.

The aims of the current paper were: first, to develop a conceptual framework for causal and effect relationships among the five dimensions of the EQ-5D instrument based on theoretical models of HRQoL, and second, to test this framework using data on EQ-5D-5L from six countries (*N* = 7933). More knowledge on the causal pattern is useful for at least two reasons: i) it provides a better understanding of the relative importance of the five health dimensions as reflected in the preference-based value sets, and; ii) it provides insights into the discussion on whether and how the QALY might be extended, e.g. by expanding the descriptive system to include additional symptom items (causal) or functioning items (effect).

## Methods

### A conceptual framework for EQ-5D dimensions

The International Classification of Functioning, Disability and Health (ICF) and the Wilson and Cleary model [11] are two recommended models for conceptualizing the relationships between dimensions in HRQoL instruments. The ICF provides a standard language and framework for describing health and health-related states and comprises two parts, each with two components [12]. Part 1 refers to functioning and disability and consists of (a) body functions and structures, and (b) activities and participation. Part 2 refers to contextual factors incorporating (a) environmental factors, and (b) personal factors. Body functions refer to physiological and psychological functions of body systems (e.g. symptoms such as pain or anxiety), while activity refers to the execution of a task or action (e.g. self-care), and participation refers to involvement in a life situation (e.g. work). The EQ-5D-3L was classified in an ICF framework [13] using linking rules [14]. Its five dimensions were classified into two ICF components, such that pain/discomfort (PD) and anxiety/depression (AD) were linked to the ICF component of body functions, while mobility (MO), self-care (SC), and usual activities (UA) were linked to the ICF component of activity and participation.

The ICF has considerable overlap with the Wilson and Cleary model [15, 16] that depicts dominant causal pathways between five levels of health outcomes: biological and physiological factors, symptoms (corresponding to the ICF component of body functions and defined as the patient’s perception of an abnormal physical, emotional or cognitive state), functioning (corresponding to the ICF component of activity and participation), general health perceptions, and overall quality of life. The Wilson and Cleary conceptual model has been empirically validated in populations with different health conditions [17–24].

Based on these models, we propose the following causal pattern between the 5 EQ-5D dimensions. Firstly, the “symptom” dimensions of pain/discomfort (PD) and anxiety/depression (AD) were assumed to be primarily causal indicators, and the “activity/participation” dimensions of mobility (MO), self-care (SC), and usual activities (UA) to be effect variables, i.e. PD and AD cause changes in the HRQoL construct that are manifested as changes in MO, SC, and UA. Physiological symptoms such as pain and discomfort are clear drivers of activity/participation items and influence walking and self-care [25, 26] and daily activities [27]. Such symptoms are likely to be unidirectional, as it is unlikely that a change in mobility or self-care would alter the level of pain experienced. We assume a predominantly causal link between AD and activity/participation (MO, SC and UA), though with AD having less influence on MO (i.e. walking) than on SC and UA, as depressive symptoms explain only a small portion of the variability in mobility scores [28]. Anxiety and depression can cause disability by worsening other symptoms or by leading to limitations in activity, e.g. lack of interest in self-care [29] and activities of daily living [30]. It was noted, however, that emotional well-being may be bidirectional [2, 15], because physical symptoms, impairments, activity limitations, or participation restrictions can cause anxiety and/or depression [29].

Secondly, we assume mobility (MO) to be both cause and effect in nature, e.g. pain/discomfort (PD) can cause limitations in MO, which in turn can cause changes in SC and UA. This places MO in an intermediate position between PD and the other two activity/participation dimensions [3, 31]. Temporal priority has further been indicated by a hierarchical onset of disability among elderly people, where problems with walking preceded problems with self-care (e.g. bathing and dressing) [32].

**Fig. 1:**
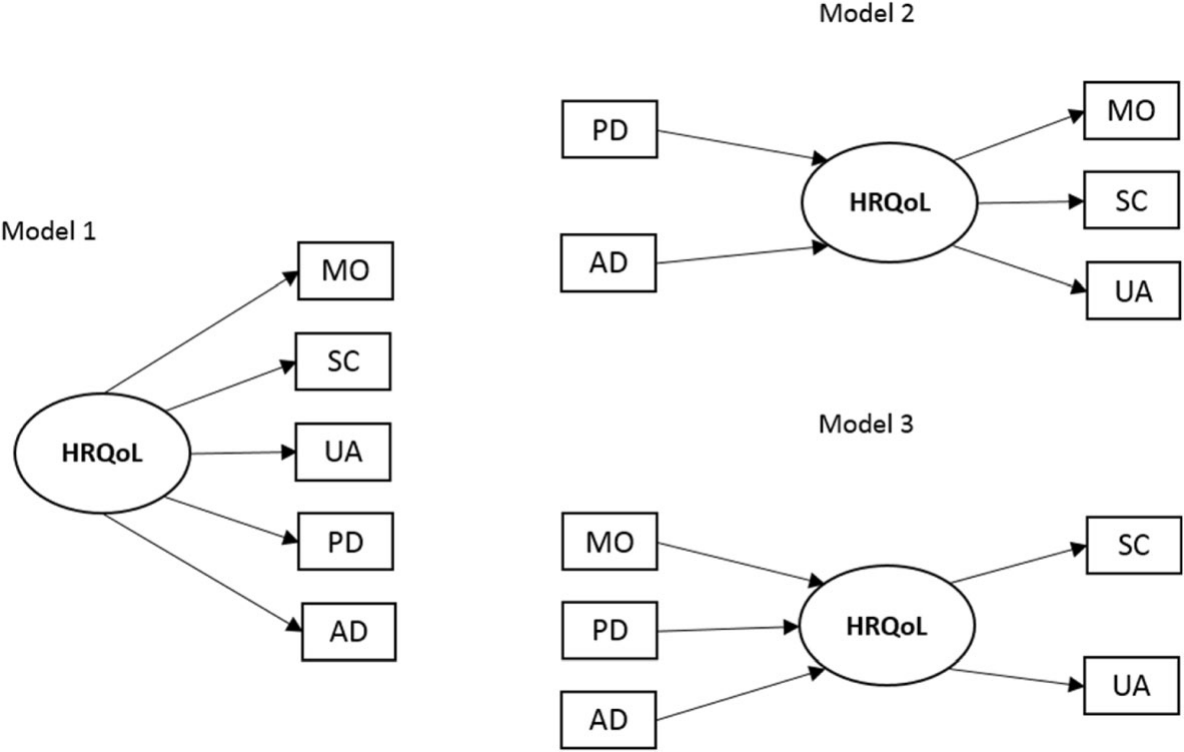
An all-effect indicator model (Model 1) and two multiple indicator multiple cause (MIMIC) models (Model 2 & Model 3). Mobility [MO], self-care [SC], usual activities [UA], pain/discomfort [PD], anxiety/depression [AD]

**Fig. 2:**
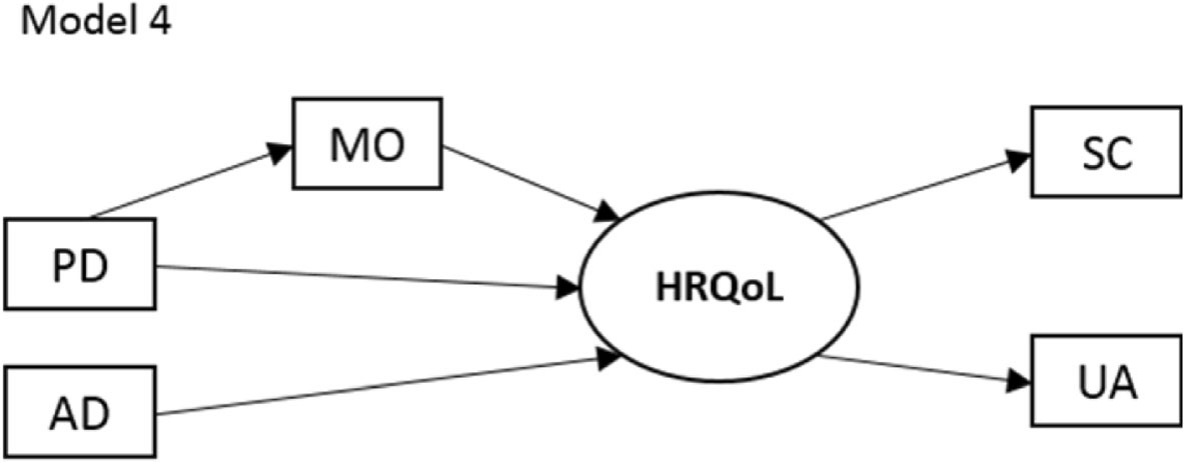
Multiple indicator multiple cause (MIMIC) model. Mobility [MO], self-care [SC], usual activities [UA], pain/discomfort [PD], anxiety/depression [AD]

Thirdly, we consider self-care (SC) and usual activities (UA) as similar dimensions that tap into activities of daily living. However, SC is more specific in that it refers to washing and dressing, while UA has a wider scope and encompasses participation in educational, employment, and social activities. Based on this conceptual framework, a number of testable models were specified (see Figs. 1 and 2) to be explained further below.

### Data

An online survey was administered in 2012 in six countries (Australia, Canada, Germany, Norway, UK, US) by a global panel company [33]. Respondents were initially asked if they had any of seven listed chronic diseases and to rate their overall health on a [0–100] visual analogue scale (VAS), where 0 represented the least desirable health and 100 represented the best possible physical, mental, and social health. Respondents qualified for the “healthy group” if they reported no chronic diseases and a VAS rating of overall health of at least 70. Respondents then completed several HRQoL instruments, including the EQ-5D-5L. Of the 7933 respondents, 6173 reported a chronic disease (arthritis, asthma, cancer, depression, diabetes, hearing loss, heart disease). For further details on respondent recruitment, see Richardson et al., 2012 [33].

### Distribution of EQ-5D health states

Spearman’s rank correlations were computed across the responses to the 5 EQ-5D dimensions. Frequency distributions of EQ-5D health states were used to examine the pattern of responses across the main distinction between symptoms (causes) vs activity/participation (effects). Two subscales were created with EQ-5D items: a Symptom subscale formed by summing the PD and AD level numbers (each from level 1 to 5), and an Activity/participation subscale formed by summing the MO, SC and UA level numbers. The relationship between the two subscales are illustrated with a graph, and descriptive statistics are provided in the Appendix.

### Structural equation modelling (SEM)

Two model-testing procedures in SEM were used: confirmatory tetrad analysis (CTA) and confirmatory factor analysis (CFA). While CTA is assumed to be the best empirical approach for determining whether items should be treated as causal or effect indicators [8], agreement between the two approaches would provide more confidence in our conceptual model than either one alone [34, 35]. While both procedures investigate the path directionality between items and an underlying construct, they both have unique features that are applicable for the current investigation. First, CFA enables testing of the hypothesised intermediate position of mobility between PD and the underlying construct, while CTA allows comparison of models that are not nested in the standard log-likelihood ratio (LR) test, but nested according to the implied vanishing tetrads (explained below).

### Confirmatory tetrad analysis

CTA seeks to determine whether items of a latent variable should be treated as causal or effect indicators [34, 36]. While a parameter estimator such as maximum likelihood (ML) method is usually applied when testing general SEM, the CTA test does not estimate parameters, but only tests model fit using Chi-square (χ^2^). The CTA test statistic depends on the tetrads produced by a model. Following Bollen and Ting [36], consider a latent variable indicated by four observed items (×_1_ – ×_4_). The effect of the latent variable to the items can be written as Eq. 1:

1 $$ {x}_i={\lambda}_{i1}{\xi}_1+{\delta}_i $$

where *δ*_*i*_ is the random measurement error (disturbance) term with Ε (*δ*_*i*_) = 0 for all i,_*,*_ *COV (δ*_*i*_, *δ*_*j*_) = 0 for i ≠ j, and COV (*ξ*_1_, *δ*_*i*_) = 0 for all i. The population covariances (σ_ij_) of the observed items are given as Eq. 2 below:

2 $$ {\upsigma}_{\mathrm{ij}}={\lambda}_{i1}{\lambda}_{j1}\phi $$

where σ_ij_ is the population covariance matrix of i and *j* items, and *ϕ* is the variance of *ξ*_1_.

A tetrad is ‘the difference between the product of a pair of covariances and the product of another pair among four random variables’ (Bollen & Ting, 2000, p.5) [34]. Thus, the four observed items produce six covariances, which can be arranged into three tetrads using Kelley’s notation [37], i.e.

3 $$ {\displaystyle \begin{array}{c}{\uptau}_{1234}={\upsigma}_{12}{\upsigma}_{34}-{\upsigma}_{13}{\upsigma}_{24}\\ {}{\uptau}_{1342}={\upsigma}_{13}{\upsigma}_{42}-{\upsigma}_{14}{\upsigma}_{32}\\ {}{\uptau}_{1423}={\upsigma}_{14}{\upsigma}_{23}-{\upsigma}_{12}{\upsigma}_{43}\end{array}} $$

where τ_ijkl_ is the population tetrad that refers to σ_ij_σ_kl_ – σ_ik_σ_jl_. If the tetrad equals to zero, that is τ_ijkl_ = 0, it is referred to as a vanishing tetrad. Hence, if the four observed items were effect indicators, the model would imply three vanishing tetrads (i.e. all tetrads in Eq. 3 should equal to 0). Furthermore, vanishing tetrads implied by a model include redundant vanishing tetrads (i.e. any two of the vanishing tetrads in Eq. 3 would imply the third) [34]. Therefore, only two vanishing tetrads are non-redundant. Redundant vanishing tetrads should be excluded from the test. This exclusion makes covariance matrix of the tetrads that is part of the test statistic non-singular, and hence its inverse will exist. For a theoretical background on the tetrad, see [36].

Regardless of the number observed items, only four random variables (e.g. σ_12,_ σ_34,_ σ_13 and_ σ_24)_ are considered at a time, and this process is repeated for all combinations of the observed items. For every foursome of items, there are three possible vanishing tetrads. Considering an all-effect model with five observed variables (e.g. one item for each of the 5 EQ-5D dimensions), there will be five different combinations of four items, and each set will have three tetrads. Thus, the model would imply 15 vanishing tetrads. We could then test the hypothesis that H^0^: τ = 0 and H^1^: τ ≠ 0 based on sample data. If the vanishing tetrads implied by the model do vanish, it would produce a good fit of the model (a non-significant χ^2^ test), which would not reject the null hypothesis. If the test were highly significant, it would favour a causal indicator structure. However, if the χ^2^ test was 0 with 0 degrees of freedom, it would indicate an all-causal indicator model (as there are no model implied non-redundant vanishing tetrads with this structure) [8].

SEM models are traditionally referred to as nested when we constrain or free a set of parameters and conduct the LR test to statistically compare models. However, some models that are not nested in parameters can be nested in terms of vanishing tetrads. That is, models are nested *‘if the model-implied non-redundant vanishing tetrads from one model are contained within the set of implied non-redundant vanishing tetrads from the other model*’ ([8], p.1532). When models are compared (i.e. nested), a χ^2^- difference test is formed, and a highly significant *p*-value would provide support for the model with fewest implied vanishing tetrads.

Three alternative models were developed for the CTA of EQ-5D dimensions (Fig. 1). Model 1 tested for any causal pattern, where all 5 EQ-5D items were treated as effect indicators, indicated by the arrows pointing away from the HRQoL construct. Models 2 and 3 are multiple cause multiple indicator (MIMIC) models: Model 2 tested whether symptom items (PD and AD) should be treated as causal indicators (indicated by the arrows pointing from the items to the HRQoL construct) and activity/participation items (MO, SC and UA) as effect indicators. Model 3 treated symptom items (PD and AD) and mobility (MO) as causal indicators, and SC and UA as effect indicators. A bootstrap tetrad test was used to minimize the problem of non-normality [38].

As explained above, an all-effect indicator model with the 5 EQ-5D items (Model 1) would imply 15 vanishing tetrads. However, a model specifying only the three activity/participation items as effect indicators (Model 2) would imply only nine vanishing tetrads (as a subset of the 15 vanishing tetrads). As illustrated in Bollen and Ting [34], this model implies nine tetrads as we always consider four random variables at a time, and any foursome of the items in Model 2 with 3 effect indicators would imply either three or one vanishing tetrads. Removing one causal indicator thus always leaves three items specified as effect indicators, whereas removing one effect indicator would always leave two items specified as effect indicators. A foursome that includes three or four effect indicators implies three vanishing tetrads (i.e. they are tetrad equivalent, which means they cannot be distinguished in terms of vanishing tetrads), while a foursome with two effect indicators implies only one vanishing tetrad. Considering Model 2 with three effect indicators and two causal indicators, the five subsets of four items would produce nine model-implied vanishing tetrads. That is, removing a casual indicator would imply three vanishing tetrads each (3 + 3). Removing an effect indicator would imply one vanishing tetrad each (1 + 1 + 1).

Following a similar procedure, Model 3 implies three vanishing tetrads. Note that a model with only one effect indicator has zero vanishing tetrads [34]. Both Model 2 and Model 3 could be compared with the all-effect indicator model with a nested CTA using χ^2^ difference test. If this test is highly significant, the model with the fewest vanishing tetrads would be favoured. In this scenario, the test is against the appropriateness of the additional vanishing tetrads implied by the all-effect indicator model. Note that models that are not nested in standard LR test can be nested in CTA. For instance, Model 3 in CTA has fewer vanishing tetrads than Model 2 and is therefore nested in Model 2. CTA is estimated using the Stata user command referred to as “tetrad” [39].

### Confirmatory factor analysis

The models in Fig. 1 can be tested using CFA. Furthermore, a MIMIC model illustrated in Fig. 2 specified the hypothesized relationships among EQ-5D dimensions where MO has an intermediate position. (Due to the uncertain nature of AD and the investigation of reversed causality, alternative models were specified, not illustrated).

Maximum likelihood (ML) estimation is considered robust when using non-continuous data [40–42] or data that violate multivariate normality assumptions [43–45]. However, since ML can be affected by deviation from normality [46], bootstrap standard errors (with 1000 bootstrap draws) were used [47]. Model fit to data was examined using fit indices, i.e. the comparative fit index (CFI), the Tucker-Lewis index (TLI), root-mean square error of approximation (RMSEA), standardized root-mean square residual (SRMR), Akaike information criterion (AIC) and sample-size adjusted Bayesian information criterion (SABIC). CFI and TLI values greater than 0.95, and SRMR less than 0.08 represent a well-fitting model [48]. While RMSEA less than 0.05 is considered to reflect a good fit [49], values as high as 0.08 reflect adequate fit [50]. AIC and SABIC are only meaningful when different models are compared, and models with the lowest values are those with the best fit.

Statistical analyses were performed in Stata version 14.0 (StataCorp LP), except the path analyses which were performed with Mplus version 6.11.

## Results

Respondent characteristics on age, sex, education, and disease groups are provided in Tables 4 and 5 in Appendix. The healthy respondents and those reporting chronic disease were similar on gender and education, but those with chronic disease were older, as could be expected. As shown in Table 1, the highest Spearman’s rank correlation were between MO and UA (0.73), while the lowest were between AD and MO (0.26), indicating support for our conceptual model. The correlation between PD and SC was lower than that between PD and MO or UA.

**Table 1:** Spearman’s rank correlations between the EQ-5D dimensions (*N* = 7933)

	MO	SC	UA	PD	AD
MO	1.00				
SC	0.57	1.00			
UA	0.73	0.59	1.00		
PD	0.63	0.42	0.62	1.00	
AD	0.26	0.27	0.40	0.35	1.00

*MO* Mobility, *SC* Self-care, *UA* Usual activities, *PD* Pain/discomfort, *AD* Anxiety/depression

**Fig. 3:**
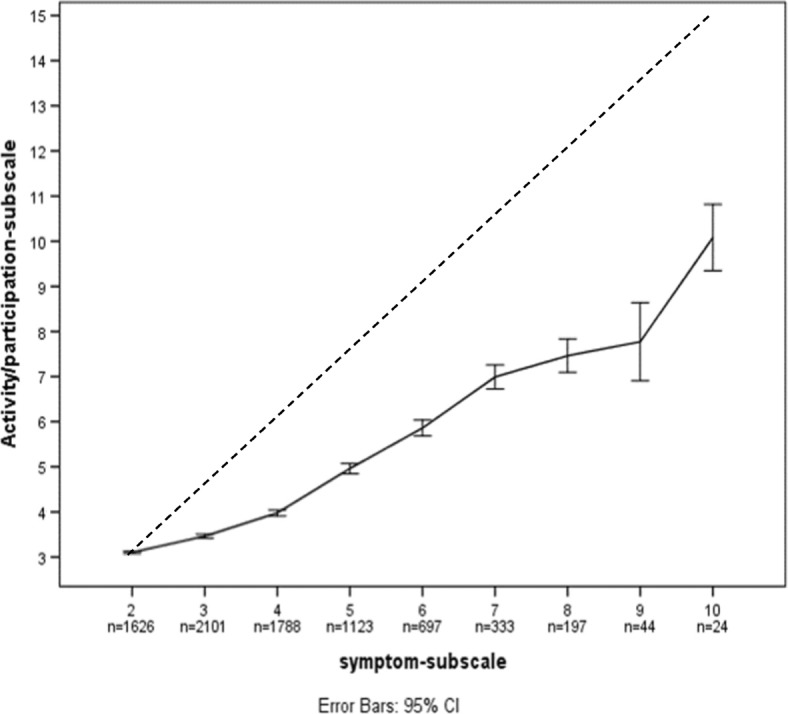
Mean summary score of effect items (MO + SC + UA) vs summary score of symptoms (PD + AD). Mobility [MO], self-care [SC], usual activities [UA], pain/discomfort [PD], anxiety/depression [AD]

Table 2 shows the frequency distribution of EQ-5D-5L health states in terms of decrements in symptom items or activities/participation items. Excluding those who reported full health (health state 11,111), the most prevalent combinations were three health states that only had slight decrements in PD and/or AD, i.e. 11121 (slight pain/discomfort), 11122 (slight pain/discomfort and slight anxiety/depression), 11112 (slight anxiety/depression). These three accounted for more than one-third (34.9%) of all possible combinations of non-perfect health states. When all health states with decrements in symptoms without any decrements in activity/participation (i.e. MO + SC + UA = 3, PD + AD > 2) were included, 47% (3031 respondents) of the sample was covered. In contrast, only 1.5% (94) of all respondents reported decrements in activity/participation without any decrements in symptoms (i.e. MO + SC + UA > 3, PD + AD = 2), suggesting that symptoms precede problems with activity/participation. Figure 3 shows the relationship between increases in the summary score of the symptom items (from 2 to 10 on the horizontal axis) and the corresponding summary score of the activity/participation items (from 3 to 15 on the vertical axis). The corresponding data are shown in Table 6 in Appendix. The results indicate that increasing pain/discomfort and anxiety/depression is associated with increasing problems with mobility, self-care and usual activities, but the problems on these activity/participation items appear to lag after the symptoms. This supports the suggestion from Table 2 that symptoms precede problems with activity/participation.

**Table 2:** Distribution of EQ-5D-5 L health states showing frequency of symptoms (pain/discomfort and anxiety/depression) vs activity/participation (mobility, self-care, and usual activities)

EQ-5D-5 L health states	N	%
11121	1135	17.7
11112	420	6.7
11122	672	10.5
1113–4,1^a^	163	2.6
11113-5	173	2.7
111, 2-5,2–5	468	7.3
Summary of MO + SC + UA = 3 and PD + AD > 2	3031	47.3
Summary of MO + SC + UA > 3 and PD + AD = 2	94	1.5
All other health states	3278	51.2
Total	6403	100.0

*MO* Mobility, *SC* Self-care, *UA* Usual activities, *PD* Pain/discomfort, *AD* Anxiety/depression

^a^11151 was not reported

The results of the CTA for Model 1 (χ^2^ = 1500.00, df = 15), Model 2 (χ^2^ = 893.79, df = 6) and Model 3 (χ^2^ = 105.84, df = 3) revealed highly significant χ^2^ estimates (*P* < 0.0001). Model 3 clearly produced the lowest χ^2^ estimates, suggesting it to be the best model. Although the significant χ^2^ estimate indicates poor fit to the data, it is usual that χ^2^ estimates are significant in large samples [51]. A nested CTA test that compared Model 2 and Model 3 revealed a highly significant χ^2^ - difference (χ^2^ diff = 787.62, df = 6, *p* < 0.0001), indicating that the model with fewest vanishing tetrads (Model 3) is favoured.

The results of the CFA are presented in Table 3. Model 1 and Model 2 produced poor fit to the data, while Model 3 produced satisfactory model fit based on CFI, TLI, RMSEA, and SRMR. These results are in line with the finding from CTA that Model 3 produced a better fit than the first two models. Model 4 (only tested with CFA) produced a satisfactory fit similar to Model 3. However, the information criteria AIC and SABIC indicate that Model 3 is the preferred one.

**Table 3:** Confirmatory factor analysis (CFA) estimates (*N* = 7933)

	Model 1	Model 2	Model 3	Model 4
CFI	0.967	0.969	0.992	0.993
TLI	0.934	0.931	0.972	0.978
RMSEA	0.117 (0.109–0.125)	0.122 (0.113–0.131)	0.075 (0.062–0.088)	0.069 (0.059–0.080)
SRMR	0.028	0.025	0.012	0.016
AIC	80,369.334	36,935.884	20,400.537	36,580.861
SABIC	80,426.349	36,977.694	20,434.746	36,626.472

*CFI* comparative fit index, *TLI* Tucker-Lewis index, *RMSEA* root-mean square error of approximation, *SRMR* standardized root-mean square residual, *AIC* Akaike information criterion, *SABIC* sample-size adjusted Bayesian information criterion

An alternative model specifying AD as an effect indicator with SC and UA did not produce a good fit, either with CTA (χ^2^ = 927.93, df = 6, *p* < 0.0001) or CFA (CFI = 0.965; TLI = 0.922; RMSEA = 0.122; SRMR 0.026). Further models investigated other specifications of the interrelationships between the three causal indicators (MO, PD and AD) in Model 4, including PD causing AD (or reversed causality), PD causing AD and MO, and PD causing AD and MO including MO as a cause of AD. All these models had a poor fit compared to the chosen model (results not reported here). The main CTA and CFA analyses were performed using the full sample (*N* = 7933), and removing the 1530 respondents reporting full health (11111) produced similar results.

## Discussion

We developed a conceptual framework for an empirical investigation of the causal and effect nature of EQ-5D dimensions. Based on theoretical models of HRQoL, the dimensions were classified as either symptoms, and thus causal, variables (PD and AD), or activities/participation and thus effect indicators (MO, SC and UA) [2, 12, 15]. While SC and UA acted as effect indicators, MO, PD and AD appeared to be causal in nature, driving changes in SC and UA. Although MO could play an intermediate role as indicated in Fig. 2, the results suggest that MO is predominantly causal.

There are reasons to believe that the role of AD might vary depending on the severity of anxiety or depression. If moderate or severe (levels 3–5), AD could reflect more of a clinical symptom that may cause dysfunctions (MO, SC, UA) and typically requires treatment. If mild (level 2), it could reflect more subjective well-being, which may vary according to personality traits (e.g. optimist vs pessimist, or level of neuroticism) and thus acts more as an effect variable (in line with the finding that emotional well-being in EORTC was an effect variable) [3]. Further investigation into the various disease groups might have indicated that the causal nature of AD is disease-specific.

Our observation of a causal pattern across EQ-5D dimensions supports the need for preference weighting [2]. The EQ-5D-5L values sets based on population preferences in four western countries (Canada, England, Spain, the Netherlands) [52–55] reveal striking similarities in the relative importance of the five dimensions. The dimensions that our conceptual model classified as causal indicators (PD and AD) have similar preference weightings, and they are on average 50% stronger than each of the three effect indicators (MO, SC, UA), i.e. the sum of the weights of the two symptom dimensions equals the sum of the three functioning items. The basis for the two causal dimensions being more important to people than the three effect dimensions might be that people find it easier to adapt to functional impairments than to pain/discomfort and anxiety/depression.

The current findings may be useful when exploring additional dimensions that could act as ‘bolt-ons’ to the five core EQ-5D dimensions. While these five dimensions have proved relevant to patients across the spectrum of diagnoses and to the general population, the EuroQol Group has been experimenting to investigate whether additional dimensions such as vision, tiredness, or sleep could enhance the instrument’s performance in some settings [56]. An interesting question is whether an HSU instrument like the EQ-5D should broaden its operationalization of the HRQoL concept in the direction of effect dimensions (e.g. social connections/network or general well-being) or in the direction of causal dimensions (e.g. vision or tiredness). Most quality of life instruments include both causal and effect indicators [57]. Causal indicators are important to measure because they affect HRQoL [2] and are often treated to avoid disruption of HRQoL. This is the rationale behind many healthcare interventions (e.g. treating arthritic pain to enable a person to continue working).

Some limitations should be acknowledged with respect to the data analyses presented here. The MIC study is based on respondents who have volunteered to participate, something which might lead to self-selection bias. Second, it is difficult to claim causality from cross-sectional data. Third, CTA is primarily intended to test for model misspecification, which does not necessarily mean that indicators are causal rather than effect indicators [35]. Future research should ideally apply panel data, which would provide better illustration of the expected temporal relationship between causal and effect dimensions.

## Conclusion

Based on theoretical models of HRQoL, we develop a conceptual framework for causal and effect relationships among the five dimensions of the EQ-5D instrument. Empirical testing on EQ-5D-5L data from a large multinational survey provided supporting evidence that the EQ-5D comprises both causal variables (Mobility, Pain/discomfort, Anxiety/depression) and effect variables (Self-care and Usual activities).

## Appendix

**Table 4:** Respondent characteristics

	Respondents (in %)		
	Healthy group	Disease groups	Total
	(*N =* 1760)	(*N =* 6173)	(*N =* 7933)
Age groups (yrs)			
18–24	11.0	5.2	6.5
25–34	17.6	10.3	11.9
35–44	18.0	13.2	14.3
45–54	19.7	21.5	21.1
55–64	15.9	27.5	24.9
65+	17.8	22.4	21.4
Education			
High School	33.6	30.6	31.3
Diploma/Certificate/Trade	38.8	40.9	40.4
University	27.6	28.5	28.3
Gender			
Female	52.2	52.2	52.2

**Table 5:** Respondents by country and disease group

Diseases	Australia	UK	USA	Canada	Norway	Germany	Total
Asthma	141	150	150	138	129	147	855
Cancer	154	137	148	138	80	115	772
Depression	146	158	168	145	140	160	917
Diabetes	168	161	168	144	143	140	924
Hearing loss	155	126	156	144	113	136	830
Arthritis	163	159	179	139	130	159	929
Heart disease	149	167	170	154	151	152	943
Healthy group	265	298	321	328	288	260	1760
Total	1341	1356	1460	1330	1174	1269	7933

**Table 6:** Mean and standard deviation (SD) of Activity/participation scale for each value on symptom scale

Symptom (PD + AD)	Activity/participation (MO + SC + UA)	SD	N
2	3.10	0.52	1614
3	3.47	1.00	2101
4	3.98	1.38	1788
5	4.96	1.98	1123
6	5.86	2.38	697
7	6.99	2.47	333
8	7.46	2.64	197
9	7.77	2.85	44
10	10.08	1.74	24

## Abbreviations

AD: Anxiety/depression
AIC: Akaike information criterion
CFA: Confirmatory factor analysis
CFI: Comparative fit index
CTA: Confirmatory tetrad analysis
HRQoL: Health-related quality of life
HSU: Health state utility
ICF: International Classification of Functioning, Disability and Health
MIMIC: Multiple cause multiple indicator model
ML: Maximum likelihood
MO: Mobility
PD: Pain/discomfort
RMSEA: Root-mean square error of approximation
SABIC: Sample-size adjusted Bayesian information criterion
SC: Self-care
SEM: Structural equation modelling
SRMR: Standardized root-mean square residual
TLI: Tucker-Lewis index
UA: Usual activities

## References

[1] Costa DS (2015). Reflective, causal, and composite indicators of quality of life: a conceptual or an empirical distinction?. Qual Life Res.

[2] Fayers PM, Hand DJ, Bjordal K, Groenvold M (1997). Causal indicators in quality of life research. Qual Life Res.

[3] Boehmer S, Luszczynska A (2006). Two kinds of items in quality of life instruments: ‘indicator and causal variables’ in the EORTC qlq-c30. Qual Life Res.

[4] Brazier J, Ratcliffe J, Salamon J, Tsuchiya A (2016). Measuring and valuing health benefits for economic evaluation.

[5] Wisloff T, Hagen G, Hamidi V, Movik E, Klemp M, Olsen JA (2014). Estimating QALY gains in applied studies: a review of cost-utility analyses published in 2010. Pharmacoeconomics.

[6] EuroQol (1990). EuroQol - a new facility for the measurement of health-related quality of life. Health Policy.

[7] Herdman M, Gudex C, Lloyd A, Janssen M, Kind P, Parkin D, Bonsel G, Badia X (2011). Development and preliminary testing of the new five-level version of EQ-5D (EQ-5D-5L). Qual Life Res.

[8] Bollen KA, Lennox RD, Dahly DL (2009). Practical application of the vanishing tetrad test for causal indicator measurement models: an example from health-related quality of life. Stat Med.

[9] Bollen K, Lennox R (1991). Conventional wisdom on measurement: a structural equation perspective. Psychol Bull.

[10] Fayers PM, Hand DJ (2002). Causal variables, indicator variables and measurement scales: an example from quality of life. J R Stat Soc A Stat Soc.

[11] Bakas T, McLennon SM, Carpenter JS, Buelow JM, Otte JL, Hanna KM, Ellett ML, Hadler KA, Welch JL (2012). Systematic review of health-related quality of life models. Health Qual Life Outcomes.

[12] WHO (2001). International classification of functioning, disability and health (ICF).

[13] Cieza A, Stucki G (2005). Content comparison of health-related quality of life (HRQOL) instruments based on the international classification of functioning, disability and health (ICF). Qual Life Res.

[14] Cieza A, Brockow T, Ewert T, Amman E, Kollerits B, Chatterji S, Ustun TB, Stucki G (2002). Linking health-status measurements to the international classification of functioning, disability and health. J Rehabil Med.

[15] Wilson IB, Cleary PD (1995). Linking clinical variables with health-related quality of life. A conceptual model of patient outcomes. Jama.

[16] Valderas JM, Alonso J (2008). Patient reported outcome measures: a model-based classification system for research and clinical practice. Qual Life Res.

[17] Chrischilles EA, Rubenstein LM, Voelker MD, Wallace RB, Rodnitzky RL (2002). Linking clinical variables to health-related quality of life in Parkinson’s disease. Parkinsonism Relat Disord.

[18] Krethong P, Jirapaet V, Jitpanya C, Sloan R (2008). A causal model of health-related quality of life in Thai patients with heart-failure. J Nurs Scholarsh.

[19] Lee DTF, Yu DSF, Woo J, Thompson DR (2005). Health-related quality of life in patients with congestive heart failure. Eur J Heart Fail.

[20] Mayo NE, Scott SC, Bayley M, Cheung A, Garland J, Jutai J, Wood-Dauphinee S (2015). Modeling health-related quality of life in people recovering from stroke. Qual Life Res.

[21] Penckofer S, Ferrans CE, Fink N, Barrett ML, Holm K (2005). Quality of life in women following coronary artery bypass graft surgery. Nurs Sci Q.

[22] Wettergren L, Björkholm M, Axdorph U, Langius-Eklöf A (2004). Determinants of health-related quality of life in long-term survivors of Hodgkin’s lymphoma. Qual Life Res.

[23] Williams KB, Gadbury-Amyot CC, Bray KK, Manne D, Collins P (1998). Oral health-related quality of life: a model for dental hygiene. J Dent Hyg.

[24] Wilson IB, Cleary PD (1996). Clinical predictors of functioning in persons with acquired immunodeficiency syndrome. Med Care.

[25] Fearon A, Neeman T, Smith P, Scarvell J, Cook J (2016). Pain, not structural impairments may explain activity limitations in people with gluteal tendinopathy or hip osteoarthritis: a cross sectional study. Gait Posture.

[26] Pollard B, Johnston M, Dieppe P (2011). Exploring the relationships between international classification of functioning, disability and health (ICF) constructs of impairment, activity limitation and participation restriction in people with osteoarthritis prior to joint replacement. BMC Musculoskelet Disord.

[27] Kose G, Hatipoglu S (2012). The effect of low back pain on the daily activities of patients with lumbar disc herniation: a Turkish military hospital experience. J Neurosci Nurs.

[28] Peel C, Sawyer Baker P, Roth DL, Brown CJ, Brodner EV, Allman RM (2005). Assessing mobility in older adults: the UAB study of aging life-space assessment. Phys Ther.

[29] Chao SF (2014). Functional disability and depressive symptoms: longitudinal effects of activity restriction, perceived stress, and social support. Aging Ment Health.

[30] Parikh RM, Robinson RG, Lipsey JR, Starkstein SE, Fedoroff J, Price TR (1990). The impact of poststroke depression on recovery in activities of daily living over a 2-year follow-up. Arch Neurol.

[31] Sullivan KJ, Cen SY (2011). Model of disablement and recovery: knowledge translation in rehabilitation research and practice. Phys Ther.

[32] Dunlop DD, Hughes SL, Manheim LM (1997). Disability in activities of daily living: patterns of change and a hierarchy of disability. Am J Public Health.

[33] Richardson J, Kahn M, Lezzi A, Maxwell A (2012). Cross-national comparison of twelve quality of life instruments: MIC paper 1: background, questions, instruments, research paper 76.

[34] Bollen KA, Ting KF (2000). A tetrad test for causal indicators. Psychol Methods.

[35] Roos JM (2014). The vanishing tetrad test: another test of model misspecification. Meas: Interdisciplinary Res Perspect.

[36] Bollen KA, Ting KF, Marsden P (1993). Confirmatory tetrad analysis. Sociological methodology.

[37] Kelley TL (1928). Crossroads in the mind of man.

[38] Johnson TR, Bodner TE (2007). A note on the use of bootstrap tetrad tests for covariance structures. Struct Equ Model Multidiscip J.

[39] Bauldry S, Bollen KA (2016). Tetrad: a set of Stata commands for confirmatory tetrad analysis. Struct Equ Model Multidiscip J.

[40] Lee S-Y, Poon W-Y, Bentler PM (1992). Structural equation models with continuous and polytomous variables. Psychometrika.

[41] Lee S-Y, Poon W-Y, Bentler PM (1990). Full maximum likelihood analysis of structural equation models with polytomous variables. Stati Probab Lett.

[42] Lee S-Y, Shi J-Q (2001). Maximum likelihood estimation of two-level latent variable models with mixed continuous and Polytomous data. Biometrics.

[43] Muthén B, Kaplan D (1985). A comparison of some methodologies for the factor analysis of non-normal Likert variables. Br J Math Stat Psychol.

[44] L-t H, Bentler PM, Kano Y (1992). Can test statistics in covariance structure analysis be trusted?. Psychol Bull.

[45] Chou C-P, Bentler PM (1995). Estimates and tests in structural equation modeling. Structural equation modeling: Concepts, issues, and applications.

[46] Nevitt J, Hancock GR (2001). Performance of bootstrapping approaches to model test statistics and parameter standard error estimation in structural equation modeling. Struct Equ Model Multidiscip J.

[47] Bollen KA, Stine RA (1992). Bootstrapping goodness-of-fit measures in structural equation models. Sociol Methods Res.

[48] Hu L-t, Bentler PM (1999). Cutoff criteria for fit indexes in covariance structure analysis: conventional criteria versus new alternatives. Struct Equ Model.

[49] MacCallum RC, Browne MW, Sugawara HM (1996). Power analysis and determination of sample size for covariance structure modeling. Psychol Methods.

[50] Browne MW, Cudeck R (1992). Alternative ways of assessing model fit. Sociol Methods Res.

[51] Vehkalahti K (2014). Structural equation modeling with Mplus: basic concepts, applications, and programming by Barbara M. Byrne. Int Stat Rev.

[52] Xie F, Pullenayegum E, Gaebel K, Bansback N, Bryan S, Ohinmaa A, Poissant L, Johnson JA (2016). A time trade-off-derived value set of the EQ-5D-5L for Canada. Med Care.

[53] Devlin N, Shah K, Feng Y, Mulhern B, Van Hout B (2018). Valuing health-related quality of life: an EQ-5D-5L value set for England. Health Econ.

[54] Ramos-Goni JM, Pinto-Prades JL, Oppe M, Cabases JM, Serrano-Aguilar P, Rivero-Arias O (2017). Valuation and modeling of EQ-5D-5L health states using a hybrid approach. Med Care.

[55] Versteegh MM, Vermeulen KM, Evers SMAA, de Wit GA, Prenger R, Stolk EA (2016). Dutch tariff for the five-level version of EQ-5D. Value Health.

[56] Devlin NJ, Brooks R (2017). EQ-5D and the EuroQol group: past, present and future. Appl Health Econ Health Policy.

[57] Fayers PM, Machin D (2015). Quality of Life : The Assessment, Analysis and Reporting of Patient-reported Outcomes.

